# Bisdemethoxycurcumin inhibits oxidative stress and antagonizes Alzheimer's disease by up‐regulating SIRT1

**DOI:** 10.1002/brb3.1655

**Published:** 2020-05-22

**Authors:** Yan Xu, Rong Hu, Duanqun He, Guijuan Zhou, Heng Wu, Chenlin Xu, Bing He, Lin Wu, Yilin Wang, Yunqian Chang, Rundong Ma, Ming Xie, Zijian Xiao

**Affiliations:** ^1^ The First Affiliated Hospital University of South China Hengyang China; ^2^ Xiangdong Hospital Hunan Normal University Zhuzhou China; ^3^ Shenzhen Baoan Shiyan People's Hospital Shenzhen China; ^4^ Xiangxi Autonomous Prefecture People's Hospital Jishou China

**Keywords:** Alzheimer's disease, bisdemethoxycurcumin, oxidative stress, silent information regulator 1

## Abstract

**Introduction:**

Alzheimer's disease (AD) is a progressive neurodegenerative disease. It can lead to progressive cognitive impairment, memory loss, and behavioral alterations. So far, the exact cellular and molecular mechanisms underlying this disorder remain unclear. And there are no effective treatments to prevent, halt, or reverse AD. In recent years, Chinese traditional medicine has become a new force in the treatment of AD, and the typical representatives of natural herbal ingredients are curcumin and its derivatives. Bisdemethoxycurcumin (BDMC), which is a classical derivative of curcumin, was found to have neuroprotective effects against a cell model of Alzheimer's disease (AD) in our previous studies. This study investigated the intrinsic mechanism of BDMC against AD in animal models.

**Methods:**

In this study, BDMC was injected into the lateral ventricles of normal C57BL/6 mice, APP/PS mice, and APP/PS mice treated with EX527 (the inhibitor of SIRT1). Y maze and Morris water maze were used to test the learning and memory ability of mice. Nissl staining was used to observe the morphological changes of neurons. Immunofluorescence staining was used to detect Aβ deposition in mice. The activities of GSH and SOD were determined to observe the levels of oxidative stress in mice. And Western blot analyses were used to detect content of SIRT1 in mice.

**Results:**

In the APP/PS mice, after BDMC intervention, their cognitive function improved, oxidative stress adjusted, the number of neurons increased, Aβ deposition decreased, and the level of SIRT1 expression increased. However, when SIRT1 is inhibited, BDMC on the improvement in the learning and memory ability and the improvement on oxidative stress in APP/PS1 mice were reversed.

**Conclusion:**

Our findings demonstrated that in the AD mice, BDMC has antagonistic effect on AD. And an intermediate step in the antagonism effect is caused by SIRT1 upregulation, which leading to decreased oxidative stress. Based on these, we concluded that BDMC injection into the lateral ventricle can act against AD by upregulating SIRT1 to antioxidative stress.

## Graphical abstract

In this work, we observed that the level of cognitive function in APP/PS1 mice is decreased and the morphology of hippocampal neurons is markedly disrupted; however, the level of cognitive function and the pathological morphology of the hippocampus in APP/PS1 mice with lateral ventricle injection of BDMC are improved. And we also found that BDMC could not antagonize AD after SIRT1 was inhibited. It provides another new direction for the development of drugs for Alzheimer's disease.

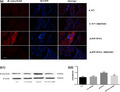

## 1 INTRODUCTION

Alzheimer's disease (AD) is a progressive neurodegenerative disease. From a pathophysiological point of view, AD is characterized by neuronal dysfunction and death, accompanied by neuroinflammation, leading to progressive cognitive impairment, memory loss, and behavioral alterations (Goldman et al., 2011). At present, the most accepted theory attributes a major role to the aberrant production and accumulation of protein aggregates, including different amyloid‐β (Aβ) peptides (Ishida et al., 2020). However, the exact cellular and molecular mechanisms underlying this disorder remain unclear (Sotolongo, Ghiso, & Rostagno, 2020) The current medications for AD are mainly cholinesterase inhibitors (including donepezil, galantamine, rivastigmine) and N‐methyl‐D‐receptor antagonists (memantine; Rygiel, 2016), which can only alleviate symptoms. There are still no effective treatments to prevent, halt, or reverse AD (Huang & Mucke, 2012). In recent years, Chinese traditional medicine has become a new force in the treatment of AD (Jiang, Gao, & Turdu, 2017), and the typical representatives of natural herbal ingredients are curcumin and its derivatives (Ahmed & Gilani, 2014).

**FIGURE 1:**
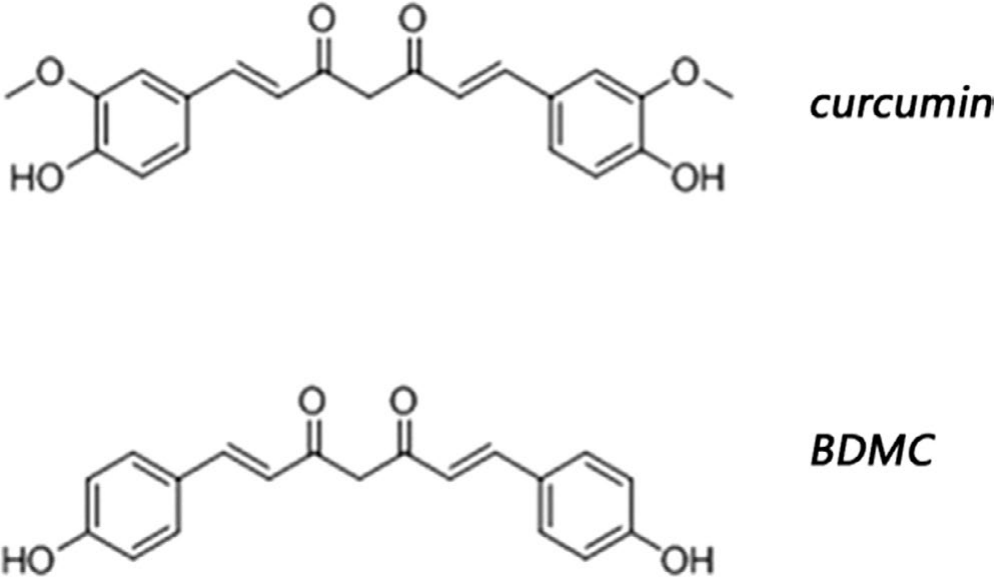
The chemical formula of curcumin and BDMC

Curcumin is a phenolic compound extracted from Curcuma longa, and it has several beneficial pharmacologic effects, including anti‐inflammatory (Derosa, Maffioli, Simental‐Mendía, Bo, & Sahebkar, 2016; Mukophadhyay, 1982), antioxidant (Uğuz, Öz, & Nazıroğlu, 2015) neuroprotective effects (Begum et al., 2008), Aβ reduction (Yang et al., 2005), and Aβ‐induced neurotoxicity (Fan et al., 2016). Despite its diverse neuroprotective properties, the use of curcumin is limited due to its low water solubility, poor absorption, and poor permeability (He et al., 2016). Consequently, there is a need to explore alternative strategies to circumvent these limitations. Studies have shown that the 3‐methoxy group on the benzene ring on both sides of curcumin inhibits the neuroprotective effect of curcumin, and the diketone structure of the main chain is an important group for neuroprotective effect. BDMC (bisdemethoxycurcumin) is a derivative of curcumin. On the basis of curcumin matrix, BDMC removes the 3‐position methoxy group on the bilateral benzene ring and retains the 4‐position hydroxyl group (Zhang, Han, Shen, Zhang, & Wang, 2019; Figure 1). It has more efficient pharmacological properties than curcumin. And our previous research in cells found that BDMC may have more potential to be a specific drug for treating AD than curcumin (Xiao et al., 2010, 2014).

Oxidative stress has been considered to be the main mediator in neurodegenerative disease and normal aging processes (Barnham, Masters, & Bush, 2004). And several studies have reported that although multiple mechanisms involve in the etiology of AD, oxidative stress seems to be the major process in AD pathophysiology (Ansari Dezfouli, Zahmatkesh, Farahmandfar, & Khodagholi, 2019). In addition, some scholars proposed the hypothesis of A beta pro‐oxidative stress for the onset of AD, which indicated that the production of A beta would lead to the increase of the level of oxidative stress in the brain of AD patients, and the increase of oxidative stress would accelerate the accumulation of amyloid peptide and tau protein in AD. Silent information regulator 1 (SIRT1) is a member of histone/non‐histone deacetylase which dependent on nicotinamide‐ademine dinucleotide (NAD+) and is currently the most popular Sirtuin protein (Yang, Jiang, Wang, & Guo, 2019). SIRT1 is attracting more and more attention for its role in resistance to oxidative stress, through mechanisms which may involve FOXOs (the mammalian forkhead transcription factors of the O class), NF‐κB, NOX (nicotinamide adenine dinucleotide phosphate‐oxidase), SOD, and endothelial nitric oxide synthase (Zhang et al., 2017). Meanwhile, there are reports that activated SIRT1 can activate its downstream targets, control oxidative stress, generate neuroprotective effect and thus play a role in controlling AD (Ma et al., 2019).

Based on the above, curcumin can be used to treat AD, and BDMC has greater medicinal value than curcumin. Meanwhile, it is reported that BDMC could effectively upregulated the expression of Sirt1 in cells (Li et al., 2013).

Therefore, in this study, we investigated whether BDMC has anti‐AD effect in vivo and explore whether BDMC antagonism to AD is related to antioxidative stress of Sirt1 pathway.

## 2 MATERIALS AND METHODS

### 2.1 Experimental animals

The APP/PS1 mice were purchased from the Animal Model Company of Guangdong Province. The C57BL/6 mice were purchased from Slack Animal Company in Changsha City of Hunan Province. The number of mice bought was evenly split between males and females, and the mice were 3 months old and had body weights of approximately 20–30 g. The mice were raised in an SPF animal room to ensure that the room temperature (24°C ±2) and humidity were suitable. The experiments were approved by the Animal Laboratory Administrative Center and the Institutional Ethics Committee of University of South China and were also in accordance with Hunan province Administration Rule of Laboratory Animals and the National Institutes of Health Guidelines.

### 2.2 The main reagent

BDMC was synthesized from curcumin purchased from ABCam. The curcumin was used as the BDMC precursor and was modified according to the method of Lijianming et al. The BCA protein quantification kit, total SOD activity detection kit (WST method), and glutathione peroxidase assay kit were purchased from Biyuntian Company.

### 2.3 Animal groups and administration

Part I experimental groups: Forty mice were randomly assigned to four groups: C57BL/6 mice + NS (*n* = 10), C57BL/6 mice + BDMC (*n* = 10), APP/PS1 mice + NS (*n* = 10), APP/PS1 mice + BDMC (*n* = 10).

Part II experimental groups: Thirty mice were randomly assigned and divided into three groups: APP/PS1 mouse group (*n* = 10), APP/PS mouse + EX527 group (*n* = 10), APP/PS mouse + EX527 +BDMC group (*n* = 10).

### 2.4 Lateral ventricle administration

For the mice used in experiment, intracranial guide cannula implantations were conducted under ketamine/xylazine (100 and 10 mg/kg, respectively) anesthesia. In the process of it, the mouse's head was fully fixed with a stereotactic locator. Stainless steel guide cannulae were inserted bilaterally 1.0 mm above the microinjection sites in the lateral ventricles (at co‐ordinates from bregma: AP: −0.2 mm, ML: ±1.0 mm, and DV: −2.0 mm with the skull horizontally flat) and secured to the skull with screws and dental acrylic. Obturators, extending 1 mm beyond the cannulae, were inserted into the cannulae to prevent blockage and remained there at all times, except during microinjections. The BDMC solution (5 µg/kg. d) was slowly injected into the lateral ventricle through a hose while the mice were in an awakened state, over the course of approximately 3 min to prevent the reverse flow of liquid and the mice were continuously injected for a month. The sterile protocol was strictly followed, and experimenters monitored intracranial infection.

### 2.5 Caudal injection

A 1 ml syringe was prepared, the mouse was secured, and importantly, the tail was straightened and tightened step. The tail was wiped with an alcoholic cotton ball or the tail was warmed with hot water or warm towels, causing the veins in the tail to expand. The mouse tail was secured. Holding 0. 1 ml in front of the 1 ml syringe. Place the right little finger on the left thumb of the mouse tail and press the needle into the hand. While injecting, the left hand pulled the tail, which injected. After the injection, medical cotton was used to stop the bleeding. All operating experiments in mice were performed according to the "Regulations on the administration of experimental animals, the guidance about animal to test regulations."

### 2.6 Morris water maze experiment

For the Morris water maze, refer to the method of Vorhees and Williams (2006) and make simple modifications, the maze consisted of a 1.2 m diameter, light blue, plastic, circular pool filled with water to a depth of 31 cm with a temperature of 22°C. The maze was divided into four quadrants (A, B, C, and D). A transparent platform was placed at the C quadrant and was submerged 1 cm beneath the surface of the water. The test included two phases: spatial navigation training and probe experiments. During spatial navigation training, mice were first placed in one of the four starting locations facing the pool wall, and were allowed to swim until finding the platform in 90 s. The time that each mouse took to find the transparent platform was recorded as the escape latency period. And it is a manifestation of spatial learning and memory acquisition ability in mice. If the mice did not find the platform within 90 s, their escape latency period was recorded at 90 s, and they were guided to the platform by the experimenter and were allowed to remain at the platform for 15 s. The process lasts five days. On day 6, the animal's latency to reach the platform tended to stabilize, the transparent platform was removed, and mice were placed in the pool at the opposite quadrant of the platform and were allowed to swim freely for 90 s. The number of times that the mouse passed through the position range of the original escape platform within 90 s, the time spent in the target quadrant and the swimming speed were used to evaluate the degree of memory consolidation.

### 2.7 Y maze

The procedure for the Y Maze did not involve any training, reward, or punishment. This test mostly assesses the learning and memory function of the hippocampus. The Y maze comprised three symmetrical arms (30 cm × 8 cm × 15 cm) separated by 120°. Every mouse was placed in the center of Y maze, with the head in the same direction, and was allowed to explore the arms freely for 5 min. Their activity was recorded for 5 min. The path of the mouse activity was recorded by software. The spontaneous alternation percentage was defined as the percentage of correct alternation times in the three arms in the total shuttle times of the three walls (correct alternation times/total shuttle times of the three arms x 100%). The higher the score, the better the learning and memory.

### 2.8 Nissl's staining

The normal paraffin‐embedded hippocampi of mice were cut into 4‐mm thick sections. After dewaxing and rehydration, the sections were stained for 30 min at 60°C with Nissl staining solution. Sections were then subjected to dehydration with anhydrous ethanol, made transparent with xylene, and sealed with neutral gum. The morphology was observed, and images were obtained using a light microscope. Statistical analysis was performed using Image Pro Plus.

### 2.9 Immunofluorescence staining

Frozen hippocampal sections were hydrated, washed with PBS, and repaired with antigen at a high temperature for 15 min. Then the sections were naturally cooled, and endogenous peroxidase blocker was added dropwise. After incubation for 20 min at room temperature, the sections were washed three times with PBS for 5 min each time. Goat serum was added dropwise and blocked at room temperature for 20 min. The serum was removed, the Aβ antibody, 6E10 (1:500, Covance, Princeton, NJ, USA. A diluent was purchased from Beyotime, China, P0103) was added and the sample was incubated overnight in a 4°C refrigerator. The next day, the samples were rewarmed at room temperature and washed with PBS three times, for 5 min each time. The secondary antibody (alexa‐647; 1:100;Cell Signaling Technology; 4414S. A diluent was purchased from Beyotime, China, P0108) was added and incubated at room temperature for 1 hr. PBS was used to rinse the sample three times, for 5 min each time. Finally, an anti‐fluorescence quenching agent was added to seal the slides. Fluorescence microscopy was used to observe and take photographs for statistical analysis.

### 2.10 Oxidative stress test

Determination of SOD and GSH activity in mouse brains: The mice were sacrificed by cervical dislocation after each group of mice underwent the behavioral tests. Then, the whole brain was weighed, RIPA lysate was added, and the sample was homogenized on ice using a glass homogenizer. After centrifugation at 111.8*g* at 4°C, the supernatant was removed. The protein concentration was determined by the BCA method. The SOD and GSH activities in the brain tissue were determined according to the kit instructions.

### 2.11 Western blot analyses

Samples were taken from the hippocampus of the mice, and then the proteins of 10 µl samples were separated by electrophoresis and transferred to polyvinylidene fluoride (PVDF) membranes. The PVDF membranes were blocked for 2 hr in a 5% nonfat formula. Then, the PVDF membranes were incubated with SIRT1 monoclonal antibody (1:1,000; Cell Signaling Technology; USA; 8469S) or anti‐beta‐amyloid 1–42 antibody (1:1,000; Abcam; British; ab201060) or β‐actin monoclonal antibody (1:5,000; Proteintech; USA;66009‐1‐Ig) overnight at 4°C. The diluent of all antibodies was TBST. On the following day, the expression of specific proteins was detected by incubating with horseradish peroxidase‐labeled secondary antibody (1:5,000; Proteintech; USA; SA00001‐1) (1:5,000; Proteintech; USA;SA00001‐2) at room temperature for 2 hr. To image with the gel imaging system, the developing liquid was added to the PVDF membrane containing the target protein, which was developed and analyzed after exposure for an appropriate time.

### 2.12 Data analysis

The analysis of differences between groups was performed using one‐way repeated measures ANOVA or LSD *t* test, which considered *p* < .05 as statistically significant. All data are presented as the mean ± standard error (mean ± *SD*) and were statistically analyzed by SPSS 21 software.

## 3 RESULTS

### 3.1 Protective effect of BDMC

#### 3.1.1 Improved learning and memory function

**FIGURE 2:**
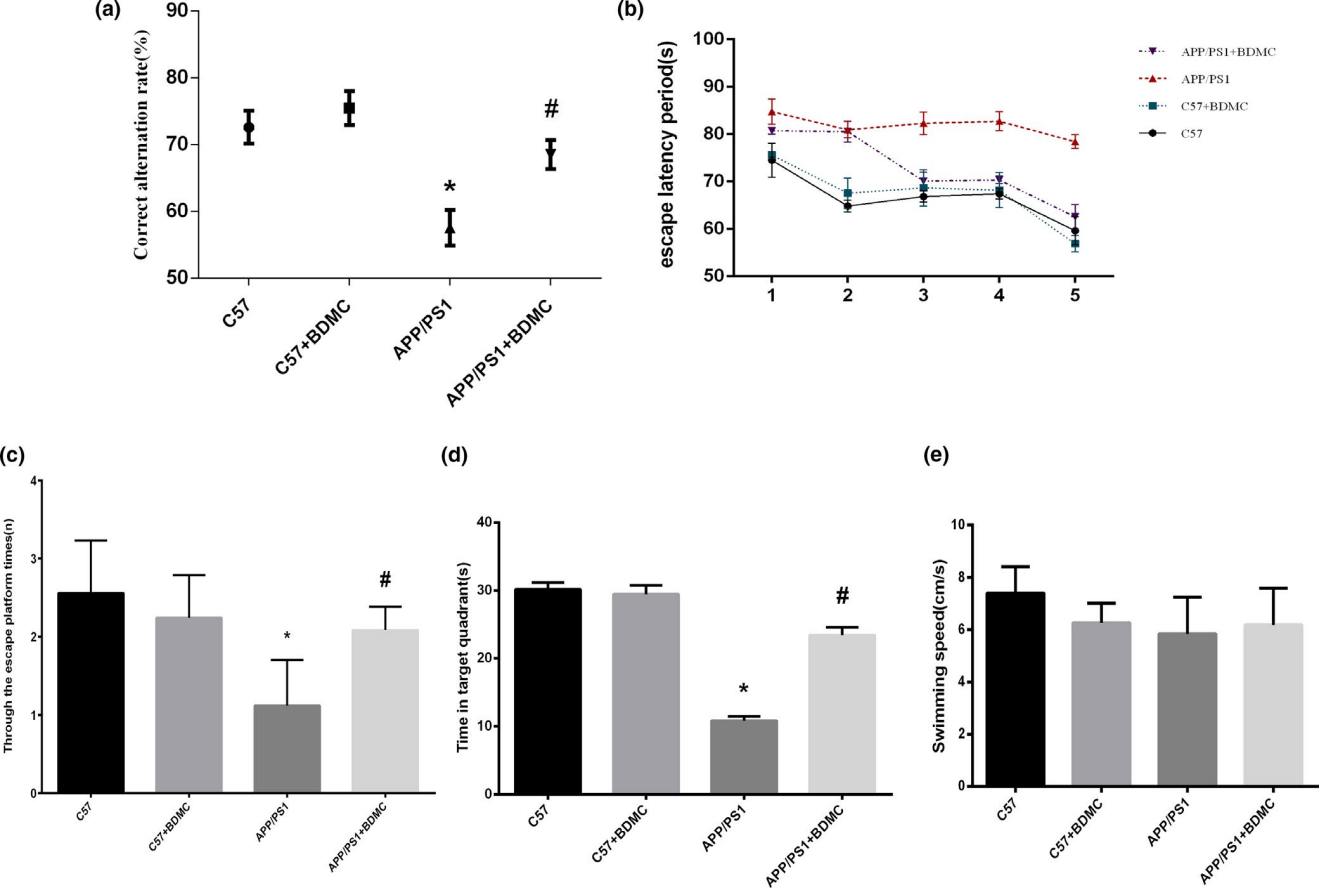
BDMC can improve learning and memory functions in APP/PS1 mice. Data are expressed as the mean ± *SD* from 5 to 7 mice per group. (a) The correct replacement rate of the mice was measured with a Y maze. (b) The escape latent period of the mice was tested with Morris water maze. (c) The mice were assayed for the number of through the escape platform region. (d) The mice were assayed for time spent in target quadrant. (e) The mice were assayed for swimming speed in Morris water maze. ^*^ *p* < .001 versus C57 mice. ^#^ *p* < .001 versus APP/PS1 mice

The Y maze and Morris water maze were used to clarify the effect of BDMC on the learning and memory ability. In the Y maze, the higher the correct alternation rate, the higher the learning and memory ability of the mice. From the Figure 2a_,_ there was no significant difference in the correct replacement rate of the C57BL/6 mice + BDMC group (72.60 ± 2.543) and the C57BL/6 mice control group (75.46 ± 2.440; *p* > .05, *p* = .1072), which suggested that BDMC had no significant effect on normal mice. The correct replacement rate of APP/PS1 mice (57.54 ± 2.645) in the Y maze was significantly lower than that in C57BL/6 mouse (*p* < .001). And after intervention with BDMC, the correct replacement rate of APP/PS1 mice (68.52 ± 2.157) went up significantly and was higher than unintervened APP/PS1 mice (*p* < .001). In the Morris water maze, during the acquisition phase, the escape latency of the APP/PS1 mice (81.84 ± 2.332) was remarkably higher than that in the C57BL/6 mice (66.65 ± 5.359; *p* < .001). After the treatment with BDMC, APP/PS1 mice (72.86 ± 7.767) improved the escape latency (*p* < .001), while the C57BL/6 mice (67.38 ± 6.719) were not affected (*p* > .05, *p* = .1925; Figure 2b). Similarly, in the probe trial, the APP/PS1 + BDMC mice (2.084 ± 0.3017) had a significantly greater number of passes across the platform than the APP/PS1 mice (1.118 ± 0.5853; *p* < .001; Figure 2c), and the APP/PS1 mice (10.82 ± 0.6535) spent much less time in the target quadrant than the APP/PS1 + BDMC mice (23.42 ± 1.154; *p* < .001; Figure 2d). In addition, in the entire MWM test, each group of mice has no difference in swimming speed (7.40 ± 1.020, 6.26 ± 0.7635,5.84 ± 1.410, 5.840 ± 1.410, 6.18 ± 1.417; C57 vs. C57 + BDMC: *p* > .05*, p* = .0804. C57 vs. APP/PS1: *p* > .05*, p* = .0799. APP/PS1 vs. APP/PS1 + BDMC: *p* > .05*, p* = .7136; Figure 2e). Taken together, our results indicated that BDMC interventions effectively improve learning and memory function in APP/PS1 mice.

#### 3.1.2 Adjusted oxidative stress

**FIGURE 3:**
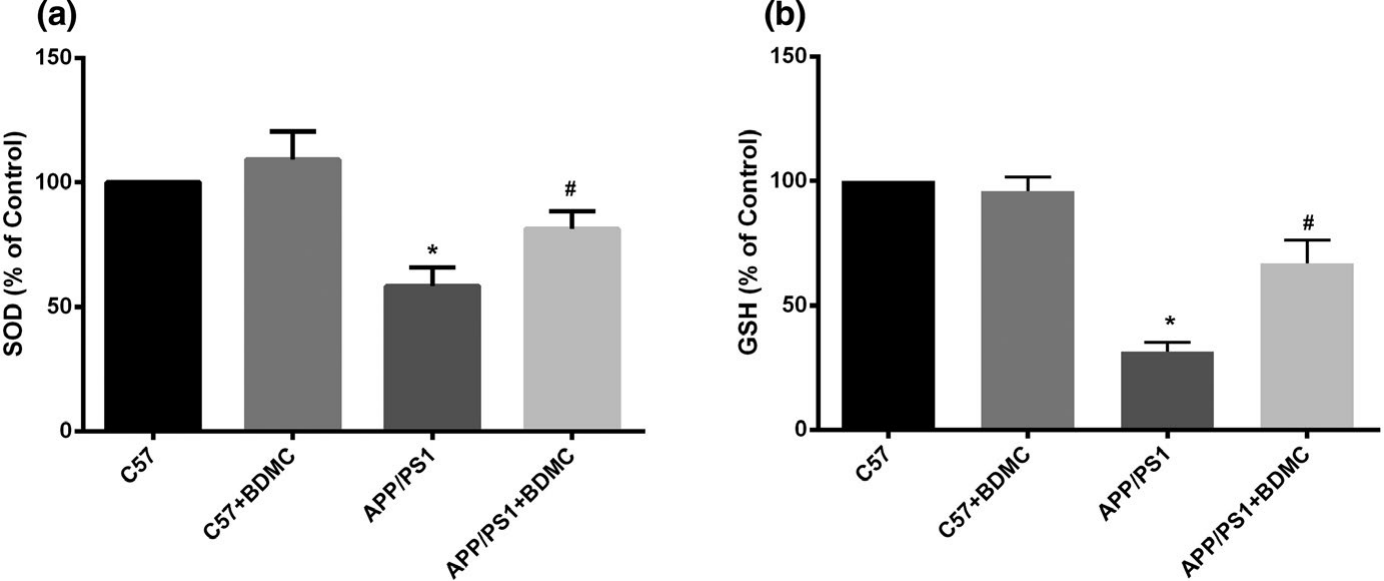
BDMC can inhibit oxidative stress in APP/PS1 mice. Data are expressed as the mean ± *SD* from 5 to 7 mice per group. (a) The activity of SOD was determined by the WST method. (b) The activity of GSH was determined with a total glutathione assay kit. ^*^ *p* < .001 versus C57 mice. ^#^ *p* < .001 versus APP/PS1 mice

SOD is one of the most important enzymes that scavenges oxygen free radicals in vivo, and the ability of the organism to remove free radicals can be evaluated by measuring the activity of SOD in vivo or in tissues. The role of GSH in the organism is to specifically catalyze the reduction of reduced glutathione to stabilize the structure of the cell membrane and protect cellular function (Zou et al., 2012). Previous studies have found that curcumin has a significant protective effect on oxidative stress (Bhattacharyya, Ghosh, & Sil, 2014). Figure 3 shows that the activity of SOD and GSH in APP/PS1 mice (58.40 ± 7.389, 31.40 ± 3.686) was lower than that in C57BL/6 mice (100.00, 100.00; *p* < .001 and *p* < .001), while BDMC significantly increased the activity of SOD and GSH in APP/PS1 mice (81.42 ± 6.887, 66.78 ± 9.502; *p* < .001 and *p* < .001). These findings indicate that BDMC could improve oxidative stress levels in APP/PS1 mice.

#### 3.1.3 Improved morphological structure

**FIGURE 4:**
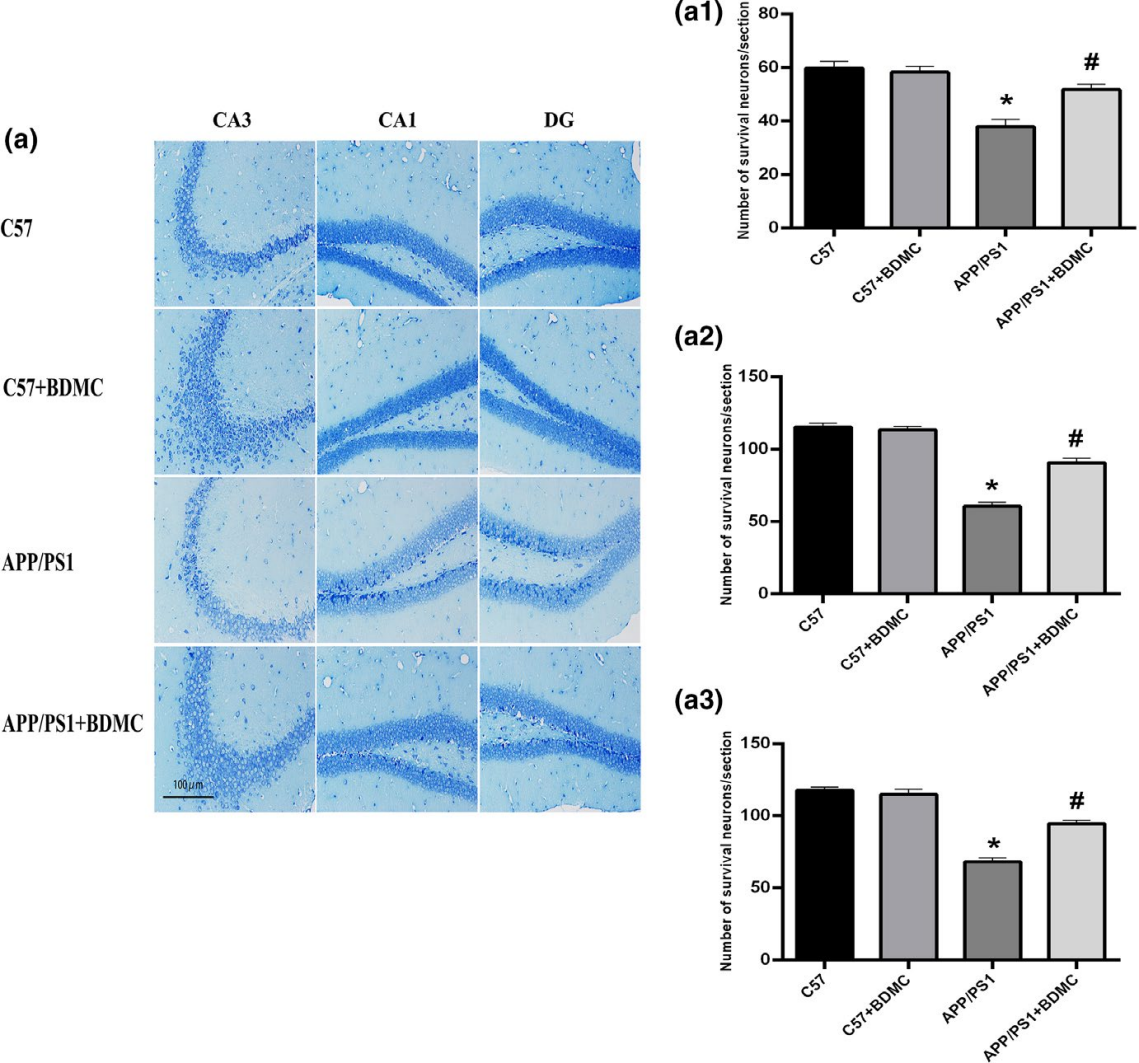
Effects of BDMC on the morphological structure of the mice hippocampus. (a) Hippocampus of Nissl staining of the 4 groups. Scale bar = 100 μm. (a_1_) Number of survival neurons of the CA3 areas. (a_2_) Number of survival neurons of the CA1 areas. (a_3_) Number of survival neurons of the DG areas. ^*^ *p* < .001 versus C57 mice. ^#^ *p* < .001 versus APP/PS1 mice

The Nissl body is the main site where neurons synthesize proteins, and it plays an important role in neuron excitation and conduction. We used Nissl staining (Figure 4) assay to observe the morphological changes of neurons and apoptosis followed BDMC treatment. The morphological results demonstrated that the hippocampal neurons of C57BL/6 mice group (In CA3 region: 59.78 ± 2.550. In CA1 region: 115.50 ± 2.523. In DG region: 117.10 ± 2.111) and C57BL/6 mice + BDMC group (In CA3 region: 58.26 ± 2.105. In CA1 region: 113.50 ± 2.415. In DG region: 114.80 ± 3.469) were not significantly different (*p* > .05, *p* = .3341, *p* > .05, *p* = .2320 and *p* > .05, *p* = .1442). However, compared with C57BL/6 mice, most hippocampal neurons were pyknotic and atrophied with irregular shape, a tangled appearance, and apoptosis cells in the hippocampus of APP/PS1 mice (In CA3 region: 37.84 ± 2.665. In CA1 region: 60.70 ± 2.686. In DG region:68.18 ± 2.700; *p* < .001, *p* < .001 and *p* < .001); after BDMC treatment (In CA3 region:51.68 ± 1.993. In CA1 region:90.64 ± 3.172. In DG region:94.56 ± 2.422), the neuronal density was increased in CA3,CA1, and DG regions (*p* < .001, *p* < .001 and *p* < .001). The results indicated that BDMC maintains APP/PS1 mice neuronal cell morphology and improves neuronal protein synthesis function.

#### 3.1.4 Decreased Aβ deposition

**FIGURE 5:**
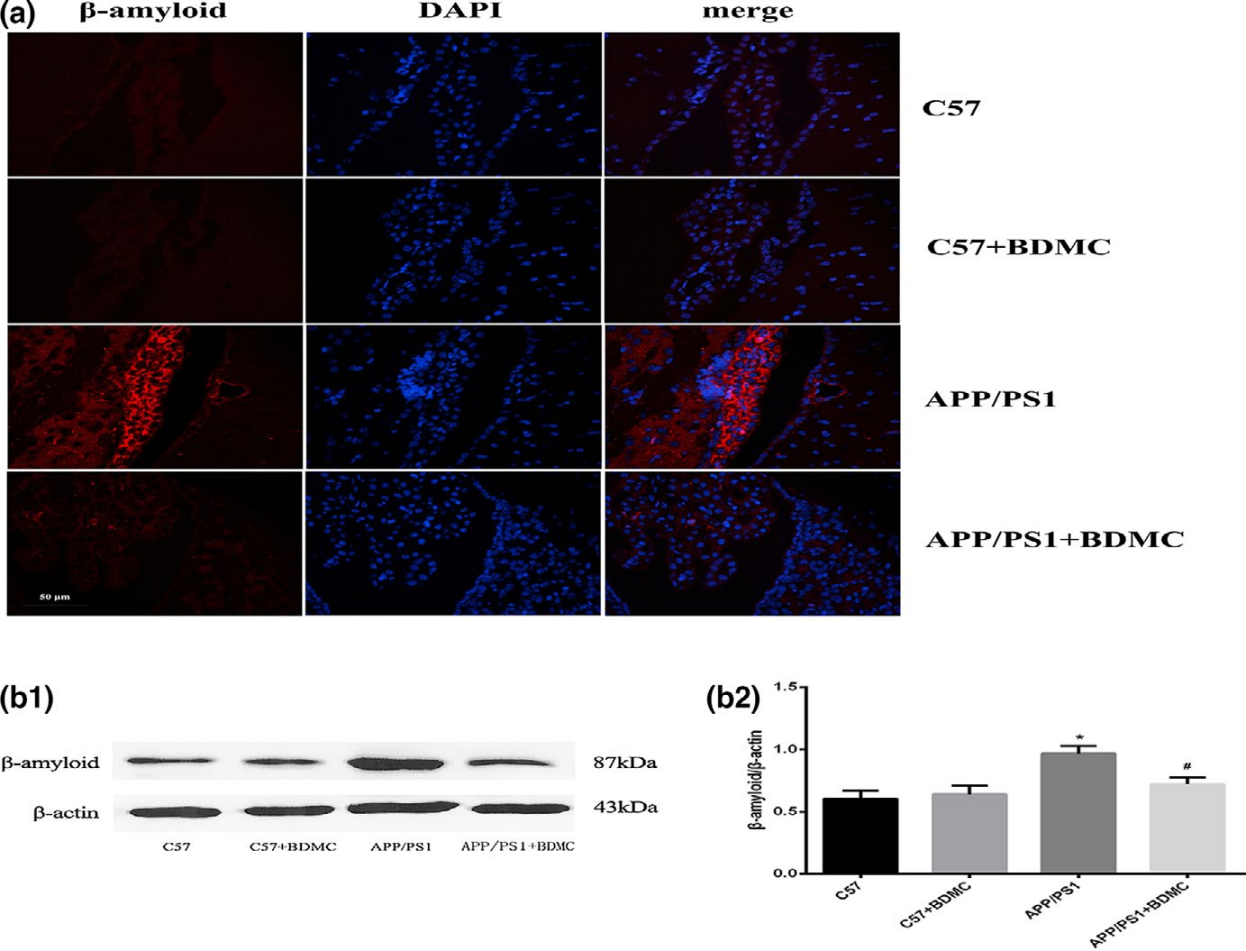
Effects of BDMC on Aβ deposition in the C57 and APP/PS1 mice hippocampus. (a) Analysis of Aβ deposition in the hippocampus with immunofluorescence staining. From left to right: red indicates the target protein Aβ, blue indicates the neuronal nucleus, and the last column shows the image after the overlay of the above two images. (b_1_) Western blot analysis. (b_2_) Quantitative analysis of b_1_ Western blot. ^*^ *p* < .001 versus C57 mice. ^#^ *p* < .001 versus APP/PS1 mice

The abnormal deposition of Aβ in the brain is one of the most important pathological hallmarks of AD, and Aβ is also a toxic substance that mainly causes nerve damage (Thal, Walter, Saido, & Fändrich, 2015). Reducing or improving Aβ toxicity is currently a key target for the prevention and treatment of AD. As shown in Figure 5a, the deposition of Aβ in the APP/PS1 mice group increased significantly compared with the C57BL/6 mice group, and the deposition of Aβ in the APP/PS1 mice + BDMC group was significantly decreased compared with that in the APP/PS1 mice group. These results indicate that the Aβ‐level of AD model mice is significantly higher than that of normal mice, which is consistent with the pathological mechanism of AD, while BDMC can significantly reduce the Aβ‐level of AD model mice. In addition, we used WB technology to further verify our results. The expression of Aβ in the APP/PS1 mice group (0.9667 ± 0.05955) increased significantly compared with the C57BL/6 mice group (0.6020 ± 0.06870; *p < *.001), and the expression of Aβ in the APP/PS1 mice + BDMC group (0.7220 ± 0.05357) was significantly decreased compared with that in the APP/PS1 mice group (*p* < .001; Figure 5b1,b2). These results indicated that BDMC could effectively inhibit Aβ deposition.

### 3.2 Effect of BDMC on SIRT1

#### 3.2.1 BDMC can upregulate the level of SIRT1

**FIGURE 6:**
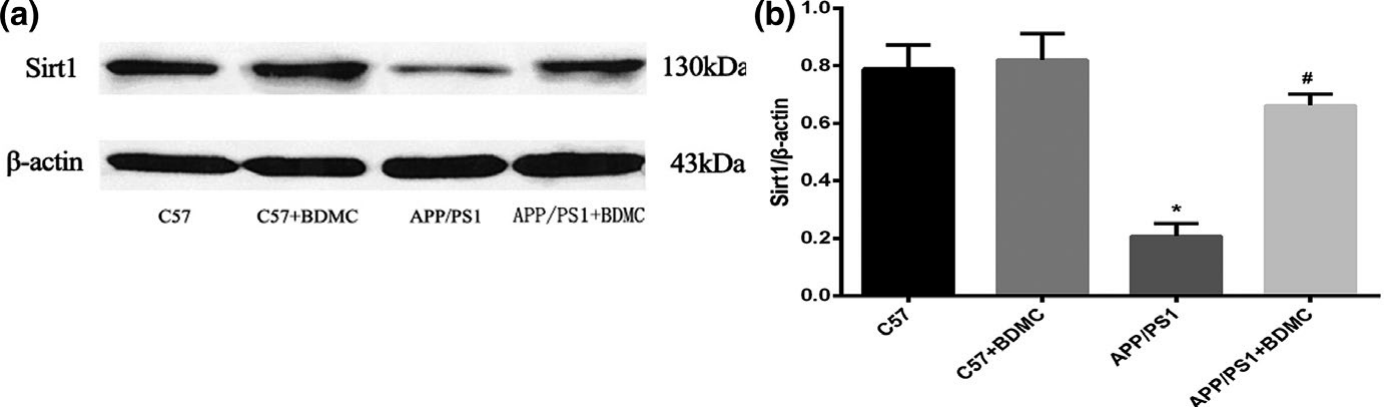
Effects of BDMC intervention on SIRT1 expression in C57 and APP/PS1 mice. (a) Quantification of SIRT1 protein expression by Western blot analysis. (b) Quantitative analysis of the Western blot results. ^*^ *p* < .001 versus C57 mice. ^#^ *p* < .001 versus APP/PS1 mice

SIRT1 is closely related to oxidative stress neuroprotection, and it has been found that the activation of SIRT1 is involved in the neuroprotective action of curcumin. In this experiment, we detected the level of SIRT1 in four groups of mice with Western blotting to determine the effect of BDMC on the level of SIRT1 in mice. The results showed that there was no significant difference in the SIRT1 levels of the C57BL/6 mice + BDMC group (0.8203 ± 0.09078) and the C57BL/6 mice control group (0.7880 ± 0.08384; *p* > .05, *p* = .5759). The SIRT1 level in the APP/PS1 mice (0.2080 ± 0.04342) was significantly lower than that in the C57BL/6 mice (*p* < .001). The level of SIRT1 in the APP/PS1 mice + BDMC group (0.6620 ± 0.03899) was significantly higher than that in the APP/PS1 mice group (*p* < .001; Figure 6), indicating that BDMC can increase the level of SIRT1 in the APP/PS1 mice.

### 3.3 EX527 can inhibit the protective effect of BDMC on APP/PS1 mice

#### 3.3.1 EX527 inhibits the expression of SIRT1

**FIGURE 7:**
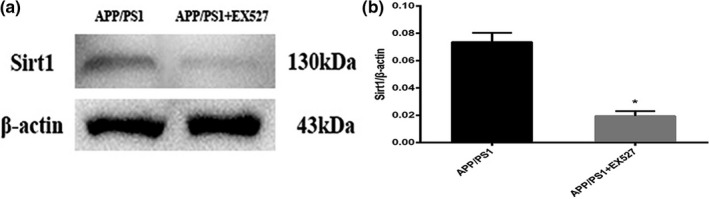
EX527 inhibits the expression of SIRT1 in APP/PS1 mice. (a) Quantification of SIRT1 protein expression by Western blot analysis. (b) Quantitative analysis of the Western blot results. ^*^ *p* < .001 versus APP/PS1 mice

The SIRT1 inhibitor EX527 was injected into APP/PS1 mice via the tail vein, and the expression of the SIRT1 protein was detected with Western blotting. The SIRT1 protein was quantified to verify the effect of EX527. Compared with the APP/PS1 mice group (0.07360 ± 0.006693), the protein levels of SIRT1 were significantly decreased in the APP/PS1 mice + EX527 group (0.01940 ± 0.003647; *p* < .001; Figure 7), which confirmed that the EX527 injection significantly inhibited the SIRT1 expression in APP/PS1 mice.

#### 3.3.2 EX527 can result in the loss of the BDMC‐mediated improvements in the learning and memory of APP/PS1 mice

**FIGURE 8:**
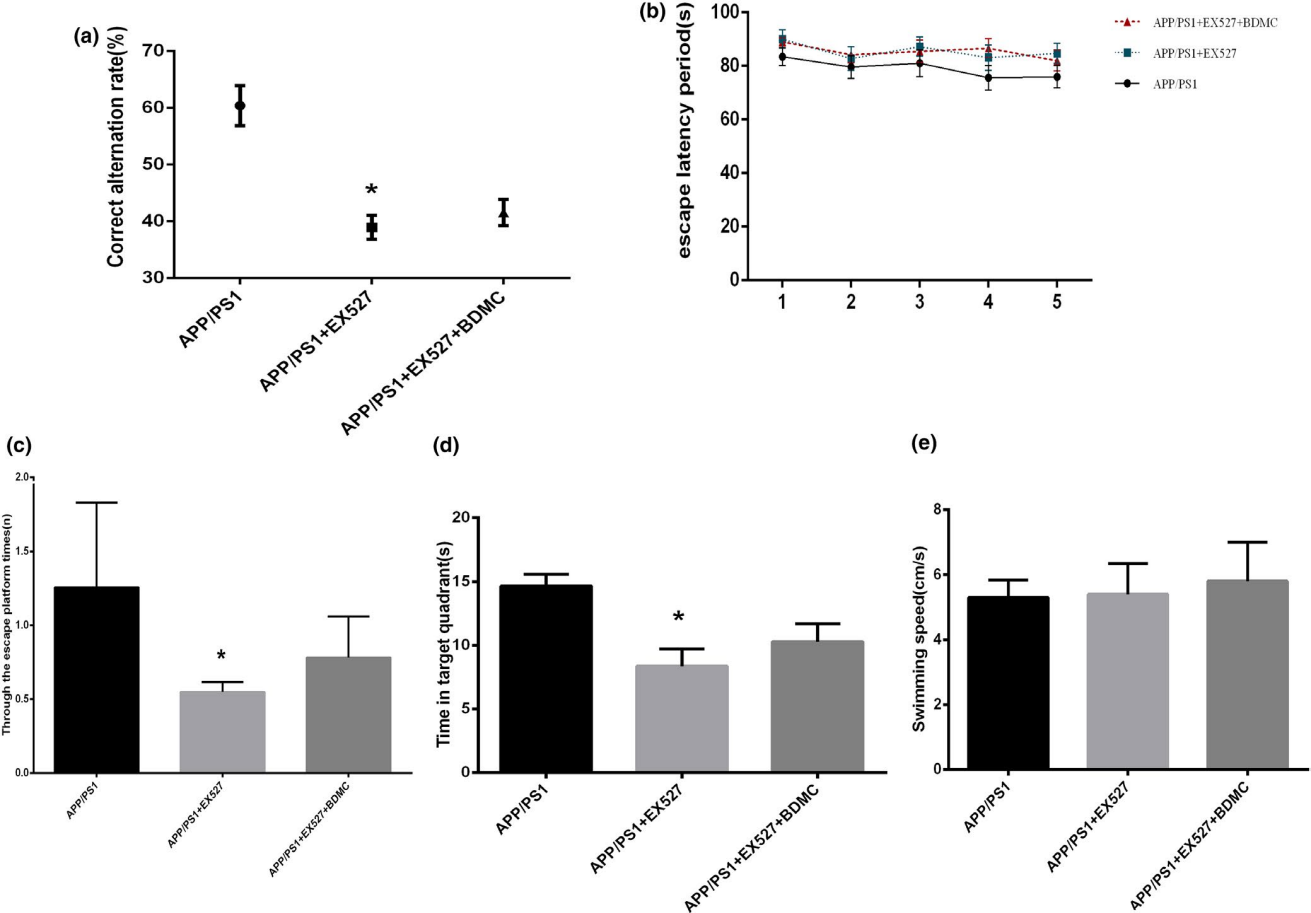
Inhibition of SIRT1 can block the BDMC‐mediated improvements in learning, memory in APP/PS1 mice. Data are expressed as the mean ± *SD* from 5 to 7 mice per group. (a) The correct replacement rate of the mice was measured with a Y maze. (b) The escape latent period of the mice was tested with Morris water maze. (c) The mice were assayed for the number of through the escape platform region. (d) The mice were assayed for time spent in target quadrant. (e) The mice were assayed for swimming speed in Morris water maze. ^*^ *p* < .001 versus APP/PS1 mice

The Y maze and Morris water maze were used again to study the learning and memory ability of experimental mice. In the Y maze, the results showed that the correct alternation rat in the APP/PS1 mice + EX527 group (38.94 ± 2.096) was lower than that of the APP/PS1 mice group (60.40 ± 3.540; *p* < .001); however, there were no significant differences between the APP/PS1 mice + EX527 + BDMC group (41.54 ± 2.302) and the APP/PS1 mice + EX527 group (*p* > .05, *p* = .988; Figure 8a). Next, during the acquisition phase, the escape latency of the APP/PS1 mice + EX527 group (85.56 ± 3.047) was longer than that in the APP/PS1 mice (79.14 ± 3.364; *p* < .001), and the BDMC intervention could not shorten the latent period of the APP/PS1 mice + EX527 group (85.46 ± 2.653; *p* > .05, *p* = .3364; Figure 8b). In the probe trial, the APP/PS1 mice + EX527 group (0.5480 ± 0.06797) had much less number of passes across the platform than the APP/PS1 mice (1.572 ± 0.4353) *p* < .001; Figure 8c), and the APP/PS1mice + EX527 mice (8.360 ± 1.346) spent much less time in the target quadrant than the APP/PS1 mice (14.66 ± 0.9209; *p* < .001; Figure 8d). Meanwhile, the intervention of BDMC had no significant effect on the improvement of the above indicators in APP/PS1 + EX527 mice (0.7820 ± 0.2769, 10.28 ± 1.417; *p* > .05, *p* = .1038 and *p* > .05, *p* = .0593). In addition, in the entire MWM test, each group of mice has no difference in swimming speed (5.300 ± 0.5385, 5.400 ± 0.9381, 5.800 ± 1.194; APP/PS1 vs. APP/PS1 + EX527: *p* > .05, *p* = .8414. APP/PS1 + EX527 vs. APP/PS1 + EX527+BDMC: *p* > .05, *p* = .5720; Figure 8e). These results indicated that the effects of BDMC on the improvement in the learning and memory ability of APP/PS1 mice can be blocked when SIRT1 is inhibited.

#### 3.3.3 EX527 can deprive the BDMC‐mediated improvement in oxidative stress in APP/PS1 mice

**FIGURE 9:**
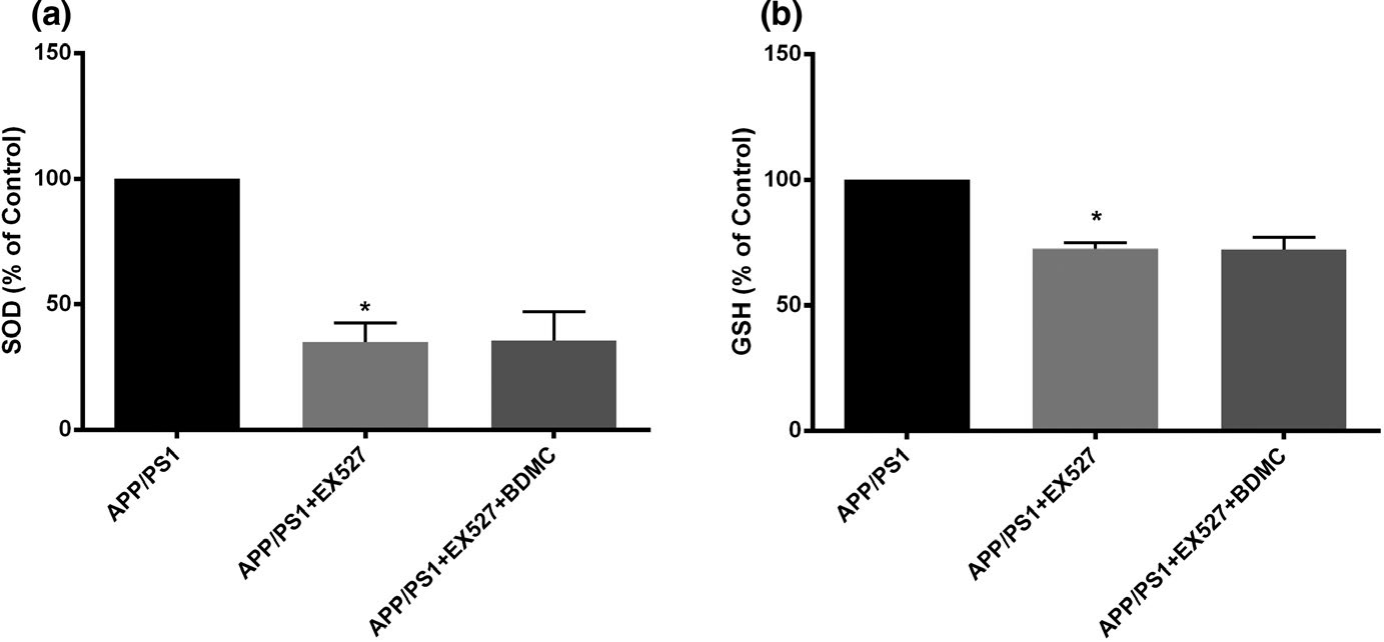
Inhibition of SIRT1 can block the BDMC‐mediated inhibition in APP/PS1 mice. Data are expressed as the mean ± *SD* from 5 to 7 mice per group. (a) The activity of SOD was determined by the WST method. (b) The activity of GSH was determined with a total glutathione assay kit. ^*^ *p* < .001 versus APP/PS1 mice

The activities of SOD in the APP/PS1 mice group, APP/PS1 mice + EX527 group, and APP/PS1 mice + EX527 + BDMC group were detected by water‐soluble tetrazolium（WST）. Clearly demonstrating the effect of SIRT1 on the inhibition of oxidative stress by BDMC, the results showed that the activity of SOD in the APP/PS1 mice + EX527 group (35.03 ± 7.639) was significantly lower than that in the APP/PS1 mice group (100.00; *p* < .001); however, there was no significant difference between the APP/PS1 mice + EX527 + BDMC group (35.74 ± 11.49) and the APP/PS1 mice + EX527 group (*p* > .05, *p* = .9117; Figure 9a). The results of SOD and GSH were similar. As the results demonstrate (Figure 9b), the GSH level of the APP/PS1 mice + EX527 group (72.45 ± 2.483) was lower than that of the APP/PS1 mice group (100.00; *p* < .001) and the APP/PS1 mice + EX527 + BDMC group (72.42 ± 4.630) was not significantly different from the APP/PS1 mice + EX527 group (*p* > .05, *p* = .9882). All of these results indicated that the SIRT1 inhibitor EX527 could reverse the effect of BDMC on oxidative stress in APP/PS1 mice.

## 4 DISCUSSION

Alzheimer's disease is an age‐related and progressive neurodegenerative disease, characterized by impaired learning and memory (Mattson, 2004). At present, researches generally start from the aspects of reducing beta‐amyloid, which causes the disease of AD (Sun, Wei‐Dong, & Yan‐Dong, 2015). APP/PS1 mice are a common animal model of AD. In our Y maze and Morris water maze, we found that the learning and memory ability of APP/PS1 mice were significantly impaired, and according to the results of the immunofluorescence staining and Western blotting methods, we also found that the Aβ levels of APP/PS1 mice increased significantly. In addition, we detected the content of SOD and GSH in APP/PS1 mice from the perspective of oxidative stress, we found that the levels of SOD and GSH in APP/PS1 mice were lower than it in wild‐type mice. All of these results indicate that the AD model is successful. In our study, we used APP/PS1 mice and C57BL/6 mice as AD models and normal control models to study whether BDMC has anti‐AD effect in vivo and to explore its possible mechanism of action.

Studies have found that curcumin can affect the cell cycle, counteract oxidative glutamate toxicity, and inhibit the effects of oxidative stress injury on AD patients (Blaylock & Maroon, 2012). However, the chemical structure of natural curcumin has some deficiencies The structure of BDMC has two fewer methoxy groups than that of curcumin, and the polarity, hydrophilicity, and water solubility of BDMC are all higher than those of curcumin (Gagliardi et al., 2012). In our vivo experiment, the Y maze and Morris water maze results showed that BDMC could significantly improve the impaired learning and memory function of AD mice. Additionally, as determined by Nissl staining, immunofluorescence staining, and Western blot analysis of Aβ, we found that BDMC could significantly increase the number of Nissl bodies and reduce the deposition of Aβ in AD mice. Based on these results, we conclude that BDMC can improve alzheimer's in vivo.

Several experimental studies have found that curcumin exerts various pharmacological effects related to the regulation of SIRT1 activity (Chung et al., 2010; Wang et al., 2014). Some studies suggested that SIRT1 is closely related to oxidative stress and upregulation of SIRT1 activity can be shown to have neuroprotective effects (Zhao et al., 2015). The oxidative stress reaction runs through the pathological process of AD. Therefore, we speculate that the mechanism by which the curcumin derivative, BDMC, effectively improves alzheimer's‐related indicators may be related to the role of sirt1 mediation In this study, by detecting the expression of SIRT1 with Western blotting, we found that SIRT1 expression was low suppressed in APP/PS1 mice, after BDMC treatment, the SIRT1 expression was increased. However, when SIRT1 was inhibited, the BDMC‐mediated improvements on AD were blocked. Therefore, we concluded that BDMC can antagonize AD by regulating SIRT1.In addition, we further examined common indicators of oxidative stress. The content of SOD and GSH in AD mice was significantly lower than that in normal mice. After BDMC intervention in APP/PS1 mice, the content of SOD and GSH increased. It suggested that BDMC can improve antioxidative stress. Next, we compared APP/PS1 mice, APP/PS1 mice + EX527, and APP/PS1 mice + EX527+BDMC. We found that BDMC could not inhibit oxidative stress after SIRT1 was inhibited. And similarly, in this situation, BDMC no longer improved the learning, memory ability, combined the results of the Y maze and Morris water maze,which suggested that the neuroprotection of BDMC was blocked following SIRT1 inhibition by EX527.

In conclusion, we observed that the level of cognitive function in APP/PS1 mice is decreased and the morphology of hippocampal neurons is markedly disrupted; however, the level of cognitive function and the pathological morphology of the hippocampus in APP/PS1 mice with lateral ventricle injection of BDMC are improved. An intermediate step in this process is caused by SIRT1 upregulation, which leading to decreased oxidative stress. Based on these,we concluded that BDMC injection into the lateral ventricle can act against AD by upregulating SIRT1 to antioxidative stress.

## CONFLICT OF INTEREST

No conflict of interest exits in the research.

## AUTHOR CONTRIBUTION

All authors contribute equally.

## Data Availability

The data that support the findings of this study are available from the corresponding author upon reasonable request.
